# Gingival Fibroblasts as a Promising Source of Induced Pluripotent Stem Cells

**DOI:** 10.1371/journal.pone.0012743

**Published:** 2010-09-14

**Authors:** Hiroshi Egusa, Keisuke Okita, Hiroki Kayashima, Guannan Yu, Sho Fukuyasu, Makio Saeki, Takuya Matsumoto, Shinya Yamanaka, Hirofumi Yatani

**Affiliations:** 1 Department of Fixed Prosthodontics, Osaka University Graduate School of Dentistry, Suita, Osaka, Japan; 2 Center for iPS Cell Research and Application (CiRA), Kyoto University, Kyoto, Japan; 3 Department of Pharmacology, Osaka University Graduate School of Dentistry, Suita, Osaka, Japan; 4 Department of Oro-Maxillofacial Regeneration, Osaka University Graduate School of Dentistry, Suita, Osaka, Japan; University of Colorado, Boulder, United States of America

## Abstract

**Background:**

Induced pluripotent stem (iPS) cells efficiently generated from accessible tissues have the potential for clinical applications. Oral gingiva, which is often resected during general dental treatments and treated as biomedical waste, is an easily obtainable tissue, and cells can be isolated from patients with minimal discomfort.

**Methodology/Principal Findings:**

We herein demonstrate iPS cell generation from adult wild-type mouse gingival fibroblasts (GFs) via introduction of four factors (Oct3/4, Sox2, Klf4 and c-Myc; GF-iPS-4F cells) or three factors (the same as GF-iPS-4F cells, but without the c-Myc oncogene; GF-iPS-3F cells) without drug selection. iPS cells were also generated from primary human gingival fibroblasts via four-factor transduction. These cells exhibited the morphology and growth properties of embryonic stem (ES) cells and expressed ES cell marker genes, with a decreased CpG methylation ratio in promoter regions of Nanog and Oct3/4. Additionally, teratoma formation assays showed ES cell-like derivation of cells and tissues representative of all three germ layers. In comparison to mouse GF-iPS-4F cells, GF-iPS-3F cells showed consistently more ES cell-like characteristics in terms of DNA methylation status and gene expression, although the reprogramming process was substantially delayed and the overall efficiency was also reduced. When transplanted into blastocysts, GF-iPS-3F cells gave rise to chimeras and contributed to the development of the germline. Notably, the four-factor reprogramming efficiency of mouse GFs was more than 7-fold higher than that of fibroblasts from tail-tips, possibly because of their high proliferative capacity.

**Conclusions/Significance:**

These results suggest that GFs from the easily obtainable gingival tissues can be readily reprogrammed into iPS cells, thus making them a promising cell source for investigating the basis of cellular reprogramming and pluripotency for future clinical applications. In addition, high-quality iPS cells were generated from mouse GFs without Myc transduction or a specific system for reprogrammed cell selection.

## Introduction

Direct reprogramming of somatic cells into induced pluripotent stem (iPS) cells by forced expression of a small number of defined factors (e.g., Oct3/4, Sox2, Klf4 and c-Myc) has great potential for tissue-specific regenerative therapies, avoiding ethical issues surrounding the use of embryonic stem (ES) cells and problems with rejection following implantation of non-autologous cells. The iPS cells have been generated from a variety of mammalian species including mice [1], monkeys [2], dogs [3], pigs [4] and humans [5]–[8]. Mouse iPS cells have been generated from cells of all three embryonic germ layers, including mesodermal fibroblasts [1] and B lymphocytes [9], endodermal hepatocytes [10], gastric epithelial cells [10] and pancreatic cells [11], and ectodermal keratinocytes [12].

The reprogramming process appears to be highly inefficient and is likely affected by many factors, including the age, type and origin of the cells used. Recently, a “stochastic model” predicted that most or all cells are competent for reprogramming [13]. However, the kinetics of reprogramming appear to vary when target populations from different tissues are used. Mouse hepatocytes and gastric epithelial cells appear to be more easily reprogrammed and require less retroviral integration than fibroblasts [10]. Dermal papilla cells, which endogenously express high levels of Sox2 and c-Myc, have been reported to be reprogrammed more efficiently than skin and embryonic fibroblasts [14]. Although the mechanisms underlying differences in reprogramming efficiency are not yet clear, some cell types might be more easily reprogrammed using specific exogenous factors than others. Importantly, the use of cell types with a high reprogramming efficiency could reduce the number of transduced factors needed, decreasing the chance of retroviral insertional mutagenesis and increasing the likelihood of ultimately replacing the remaining factors with small molecules [15]. For future clinical application, it is therefore crucial to identify cell types that can be more easily reprogrammed; ideally, these cells should also be derived from a feasible and accessible source tissue to permit autologous use.

From the standpoint of accessibility, the oral mucosa is one of the most convenient tissues for biopsy. Indeed, gingival tissues are routinely resected during general dental treatments, such as tooth extraction, periodontal surgery and dental implantation, and are generally treated as biomedical waste. Interestingly, clinical observations and experimental animal studies consistently indicate that wound healing in the oral mucosa has better outcomes than in the skin [16], [17], although the healing process and sequence are similar. Therefore, it has been postulated that oral mucosal cells possess distinctive characteristics promoting accelerated wound closure [18], [19]. The oral mucosa is composed of a thin keratinocyte layer with underlying connective tissue. Gingival fibroblasts (GFs), which are the major constituents of the gingival connective tissue, play an important role in oral wound healing, and are phenotypically and functionally different from skin fibroblasts [18]–[20]. The establishment of primary GF cultures is relatively simple because GFs adhere and spread well on culture plates, and proliferate well without requiring specific culture conditions [21].

Stem cell-based therapies using bone marrow aspirates have been successfully used in dentistry to regenerate maxillary/mandibular bones and periodontal tissue [22]–[25]; however, bone marrow aspiration from iliac crest is not an easy operation for dentists because of limitations of the dental license and specialty. Efficient reprogramming of GFs could make the gingiva an ideal source for iPS cells that could be used for autologous cell therapy and drug screening applications, especially in dentistry. In addition, gingiva normally discarded as biomedical waste would be an ideal source of donor cells from healthy volunteers to establish an iPS cell bank for a wide range of medical applications. We hypothesized that iPS cells could be produced from fibroblasts derived from gingival tissue. Such cells could be used as a valuable experimental tool for investigating the basis of cellular reprogramming and pluripotency, with possible future clinical applications.

## Results

### Generation of Mouse GF-Derived iPS Cells

**Figure 1:**
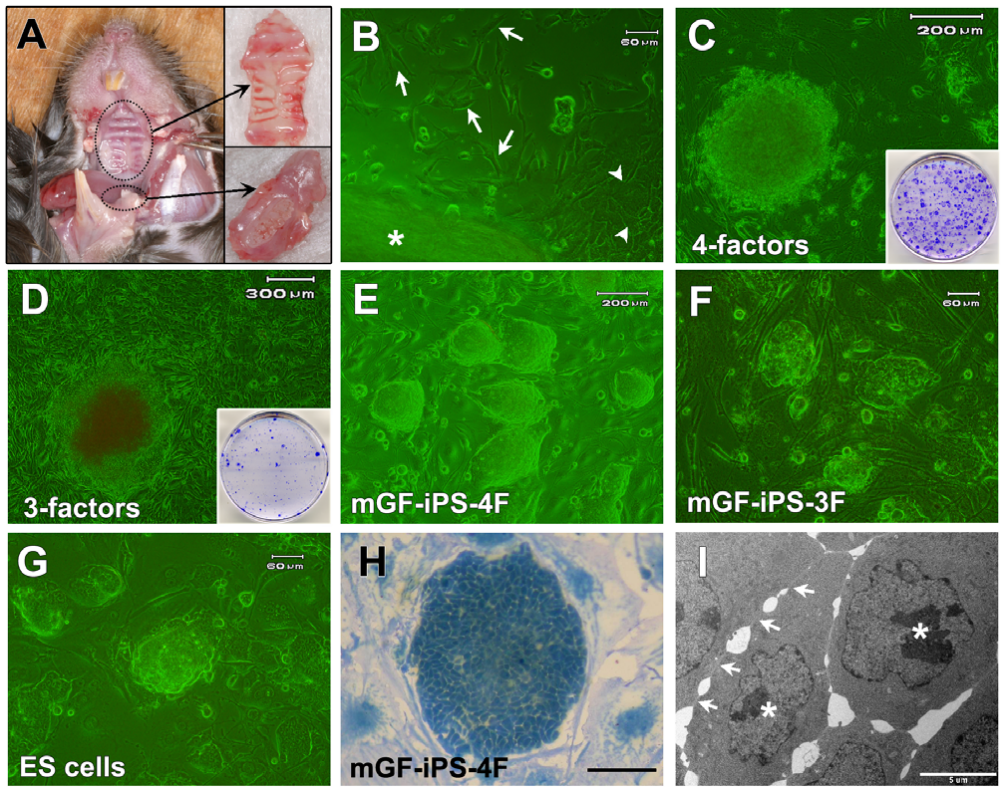
Generation of mouse GF-derived iPS cells. (A) Palatal (upper inset) and mandibular (lower inset) gingival tissues of adult mice were extracted for establishment of primary GFs. (B) Fibroblasts (arrow) and epithelial cells (arrowhead) migrated out of the palatal gingival tissue (asterisk). Scale bar; 60 µm. (C) The morphology of a colony derived from mGFs 19 days after transduction of the four factors. Scale bar; 200 µm. Inset: Colonies in a 60-mm dish after staining with crystal violet (CV) on day 21. (D) The morphology of a colony derived from mGFs 49 days after transduction of the three factors. Scale bar; 300 µm. Inset: Colonies in a 100-mm dish after staining with CV on day 50. (E–G) Morphology of (E) mGF-iPS-4F-1 cells (Scale bar; 200 µm), (F) mGF-iPS-3F-1 cells (Scale bar; 60 µm) and (G) mouse ES cell line (Scale bar; 60 µm). (H) Morphology of a pGF-iPS-4F-1 colony stained with methylene blue. Scale bar; 50 µm (I) TEM photograph of a pGF-iPS-4F-1 cell colony showing tight and continual cell membrane contacts (arrows), large nucleoi (asterisks) and scant cytoplasm. Scale bar; 5 µm.

Mouse GF cultures were established from either palatal mucosal tissues (pGFs) or mandibular tissues (mGFs) obtained from adult male mice (**Fig. 1A, 1B**). After four-factor transduction, several small-cell colonies were detected in pGFs and mGFs cultures (5 passages) under phase contrast microscopy within 14 days (10 days on feeders). More than 100 colonies were obtained in each 60-mm dish of pGF (day 17) and mGF (day 21) cultures (**Fig. 1C**). GFP expression in the cultures was monitored under fluorescence microscopy because the correct generation of iPS cells requires the silencing of the retroviral transgenes [26]. Ten colonies from each dish (total 20 colonies) showing lower GFP expression were picked up for further expansion in ES medium. After expansion, 5 clones (3 from pGF cultures and 2 from mGF cultures: **Fig. 1E**) displaying proliferation and morphology characteristic of ES cells (**Fig. 1G**) were selected.

For three-factor transduction, a small number (approximately 50 in a 100-mm dish) of ES cell-like colonies emerged in both the pGF and mGF cultures with few background cells within 50 days after transduction (**Fig. 1D**). Twenty colonies in the mGF culture were then mechanically picked for expansion. Most colonies were expandable, and ten colonies were finally selected for clonal iPS cell cultures (**Fig. 1F**).

The colonies in the selected clone cultures grew in a tight and round shape (**Fig. 1H**). Transmission electron microscopy (TEM) revealed tight and continual cell membrane contacts, and showed a large nuclear to cytoplasmic ratio and prominent nucleoli (**Fig. 1I**), representing the typical ultrastructure of mouse ES and iPS cells [27]. We refer to these ES-like cells as pGF-iPS-4F-1, -2, -3 and mGF-iPS-4F-1, -2 cells (four-factor transduction), and mGF-iPS-3F-1 to -10 cells (three-factor transduction).

### Characteristics of Mouse GF-iPS Cells

**Figure 2:**
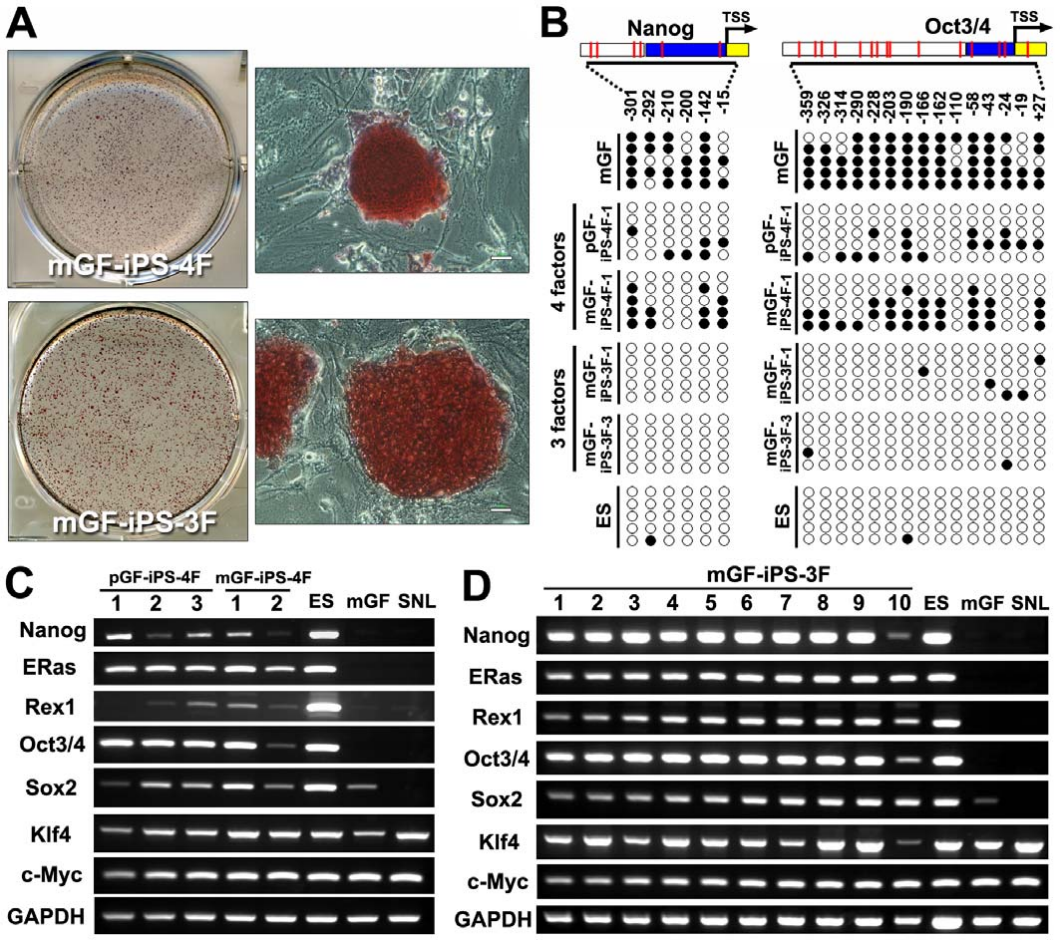
Characteristics of the mouse GF-iPS cells. (A) GF-iPS cell colonies (upper panels: pGF-iPS-4F-1, lower panels: mGF-iPS-3F-4) positively stained for ALP. Scale bar; 30 µm. (B) Bisulfite sequencing of the Nanog and Oct3/4 promoters revealed that CpGs in the parental mGFs were converted to a demethylated state in the GF-iPS cells induced by the three or four factors, resulting in a methylation pattern similar to that of mouse ES cells. The numbers in the panel indicate CpG loci respective to the transcription start site (TSS) of the genes (blue: untranslated region, yellow: translated region). (C and D) RT-PCR analysis of ES cell marker genes (Nanog, ERas, Rex1) and endogenous Oct3/4, Sox2, Klf4 and c-Myc genes in GF-iPS-4F clones (C) or GF-iPS-3F clones (D), mouse ES cells, parental mGFs and SNL feeder cells. GAPDH was used as a loading control.

All generated GF-iPS-3F and -4F cell colonies showed robust staining for alkaline phosphatase (ALP) (**Fig. 2A**). To confirm ES cell-like characteristics, the expression of undifferentiated ES marker genes was determined by reverse transcription-polymerase chain reaction (RT-PCR). All GF-iPS-4F cell clones expressed various markers for undifferentiated ES cells, including Nanog, ERas, Rex1 (Zfp42), and Oct3/4 (endogenous), to various extents but at lower levels than in mouse ES cells (**Fig. 2C**). All GF-iPS-3F cell clones expressed these ES cell marker genes at levels comparable to those in ES cells (**Fig. 2D**). In contrast, these genes were not expressed in parental GFs and SNLP feeder cells.

Bisulfite genomic sequencing was performed to evaluate the methylation status of cytosine guanine dinucleotides (CpGs) in the promoter regions of the pluripotency-associated genes, Nanog and Oct3/4. The methylation analysis revealed the percentage methylation of CpGs in the Nanog promoter regions of mGF, GF-iPS-4F (average of 2 clones), GF-iPS-3F (average of 2 clones) and ES cells to be 73.3%, 30%, 0% and 3.3%, respectively. The respective percentages for Oct3/4 in mGF, GF-iPS-4F (average of 2 clones), GF-iPS-3F (average of 2 clones) and ES cells were 85.3%, 30.7%, 4.7% and 1.3% (**Fig. 2B**). These results suggest that the highly methylated CpGs in Nanog and Oct3/4 promoters of parental GF cells were demethylated, and that these promoters became active during iPS cell induction.

### Differentiation of Mouse GF-iPS Cells

**Figure 3:**
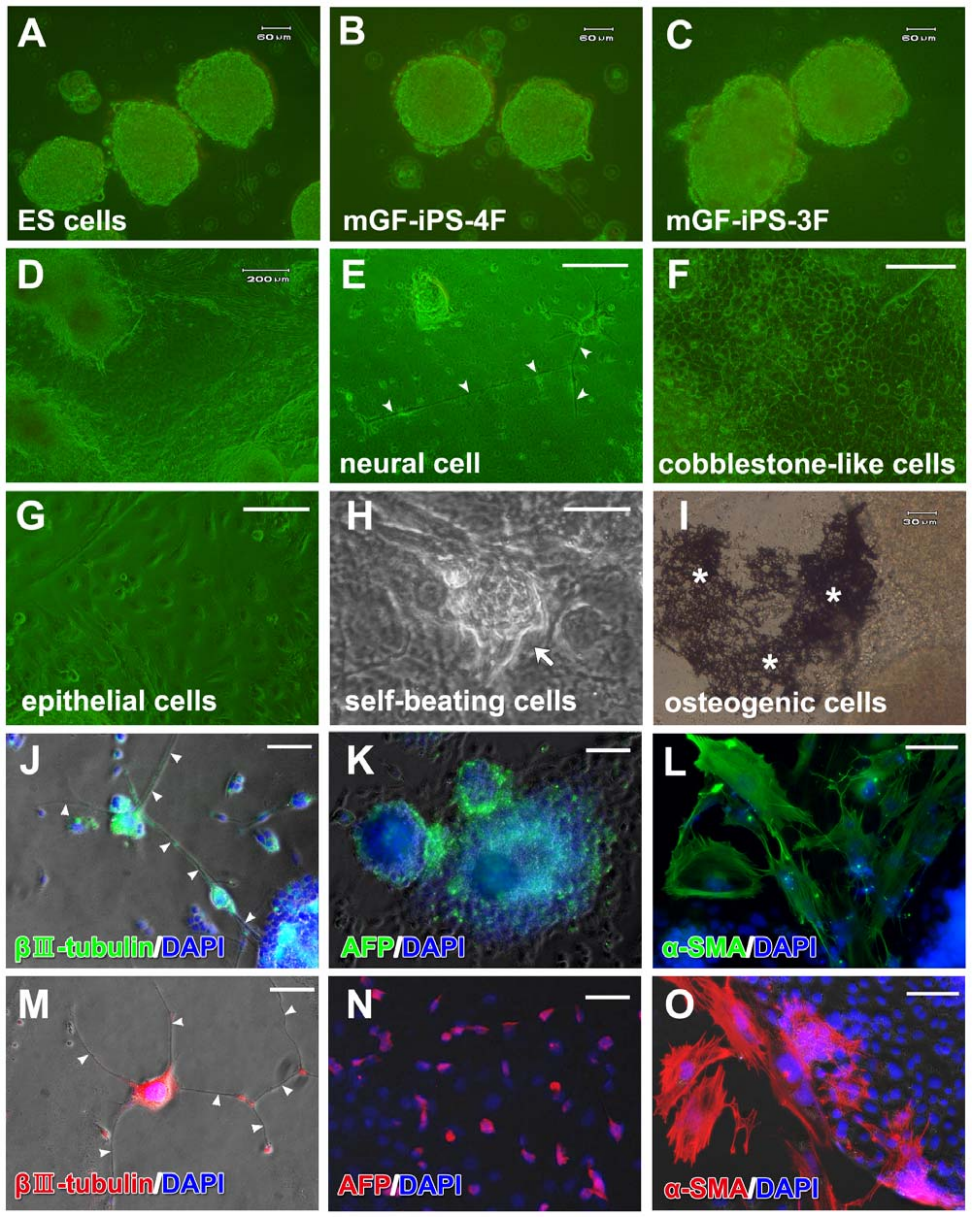
*In vitro* differentiation of mouse GF-iPS cells. (A–C) *In vitro* EB formation of (A) mouse ES cells, (B) pGF-iPS-4F-2 cells and (C) mGF-iPS-3F-9 cells after 3 days in floating culture. Scale bars; 60 µm. (D) Morphology of pGF-iPS-4F-3 cells cultured on gelatin-coated plates without feeder cells for 10 days. Scale bar; 200 µm. Attached cells showed various morphologies, such as those resembling (E) neural cells (arrowheads: neurite-like outgrowth), (F) cobblestone-like cells and (G) epithelial cells. Scale bars; 100 µm. (H) Beating myocardial cells (arrow) in the pGF-iPS-4F-3 culture after 20 days of expansion. Scale bar; 100 µm. (I) Osteogenic cells with mineralized nodule formation (asterisks) in the pGF-iPS-4F-1 cell culture detected by von Kossa staining after 30 days of expansion. Scale bar; 30 µm. (J–L) pGF-iPS-4F-1 cells and (M–O) mGF-iPS-3F-2 cells were specifically directed to differentiate into cells from all three germ layers at days three and ten after expansion, respectively. (J and M) β-III tubulin-positive ectodermal neural cells (arrowheads: neurite-like outgrowth). (K and N) AFP-positive endodermal hepatic cells. (L and O) α-SMA-positive mesodermal smooth muscle cells. Nuclei are stained with DAPI. Scale bars; 200 µm.

After 3 days of floating cultivation, the GF-iPS cells and mouse ES cells formed ball-shaped structures and embryoid bodies (EBs) (**Fig. 3A–C**). Ten days after the expansion of EB-like structures from pGF-iPS-4F cells on gelatin-coated plates, the attached cells showed various morphologies (**Fig. 3D**), resembling neuronal cells, cobblestone-like cells, and epithelial cells (**Fig. 3E–G**). By twenty days after expansion, some clumps of cells had started pulsating, suggesting that they had differentiated into cardiomyocytes (**Fig. 3H** **and Movie S1**). Thirty days after expansion, von Kossa staining revealed osteogenic cells with mineralized nodule formation (**Fig. 3I**). Immunocytochemistry revealed positive staining for β-III tubulin (a marker of ectoderm), α1-fetoprotein (AFP) (endoderm) and α-smooth muscle actin (α-SMA) (mesoderm) in pGF-iPS-4F-1 (**Fig. 3J–L**) and mGF-iPS-3F-2 cell cultures (**Fig. 3M–O**). Other clones of GF-iPS-4F and -3F cells also showed positive staining for these proteins. These data demonstrate that GF-iPS-4F and GF-iPS-3F cells could differentiate into cells from all three germ layers *in vitro*.

### Teratoma Formation by Mouse GF-Derived iPS Cells

**Figure 4:**
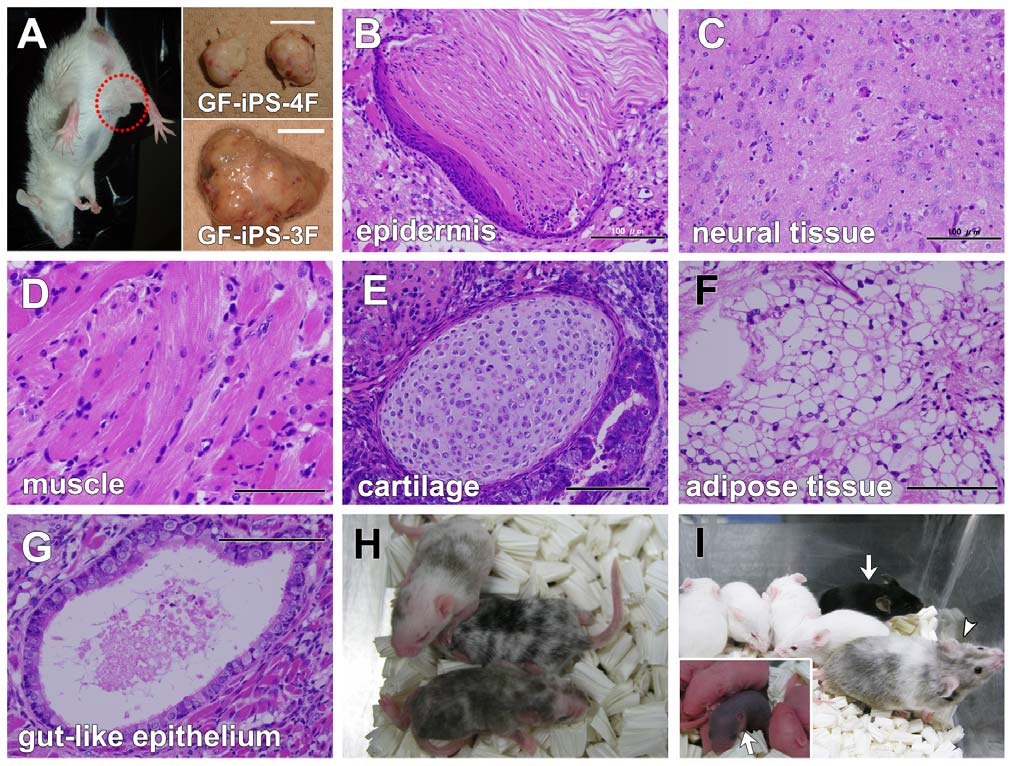
Teratoma formation and germline chimeras from mouse GF-iPS cells. (A) Transplantation of mGF-iPS-3F-1 and pGF-iPS-4F-1 cells into mouse testes resulted in apparent teratoma formation (dotted circle: tumor formation by pGF-iPS-4F-1 cell transplantation at week 10). Insets: Extracted teratomas from mice transplanted with pGF-iPS-4F-1 (upper) or mGF-iPS-3F-1 (lower) cells. Scale bars; 1 cm. (B–G) H&E staining of teratoma sections showed differentiation of mGF-iPS-3F-1 cells into various tissues from all three germ layers, including keratin-containing epidermal tissues (B: ectoderm), neural tissues (C: ectoderm), striated muscle (D: mesoderm), cartilage (E: mesoderm), adipose tissues (F: mesoderm) and gut-like epithelial tissues (G: endoderm). Scale bars; 100 µm. (H) Chimeric mice generated by injecting the black mouse-derived mGF-iPS-3F-1 cells into white mouse-derived blastocyst embryos. (I) An adult old chimeric male mouse generated from mGF-iPS-3F-3 cells (arrow head) was mated with Jcl:MCH white female mice and achieved germline transmission, as indicated by coat color in black (arrow). Inset: A newborn mouse generated via germline transmission (arrow).

Apparent tumor formation was observed in mGF-iPS-3F- and pGF-iPS-4F-injected mice at weeks seven and ten after injection, respectively (**Fig. 4A**). Extracted tumors containing GF-iPS-3F cells were larger than those containing GF-iPS-4F cells (**Fig. 4A**). Histological examinations showed that the tumors contained various tissues (**Fig. 4B–G**), including keratin-containing epidermal tissues (ectoderm), neural tissues (ectoderm), striated muscle (mesoderm), cartilage (mesoderm) and gut-like epithelial tissues (endoderm). These data demonstrate that the GF-iPS-3F and -4F cells generated in our study were capable of differentiating into tissues representative of the three germ layers *in vivo*.

### Germline Chimeras from GF-iPS-3F Cells

GF-iPS-3F cells (C57BL/6J black mouse-derived) were microinjected into Jcl:MCH white mouse-derived blastocysts, which were then transplanted into the uteri of pseudo-pregnant Jcl:ICR white mice. This yielded 13 out of 52 (25%), 9 out of 22 (40.9%), 21 out of 34 (61.8%), 19 out of 22 (86.4%) and 16 out of 27 (59.3%) adult chimeric mice from mGF-iPS-3F-1, -2 -3, -4 and -6 cells, respectively, as determined by the coat color (**Fig. 4H**).

Chimeric mice from the mGF-iPS-3F-3 clone were then mated with Jcl:MCH white females to verify germline transmission, and one pup obtained from the mating was derived from mGF-iPS-3F cells, as revealed by coat color in black (**Fig. 4I**). Taken together, these data demonstrate that GF-iPS-3F cells possess *in vivo* developmental potential comparable to that of ES cells.

### Reprogramming Efficiency of Mouse GF-Derived iPS Cells

**Figure 5:**
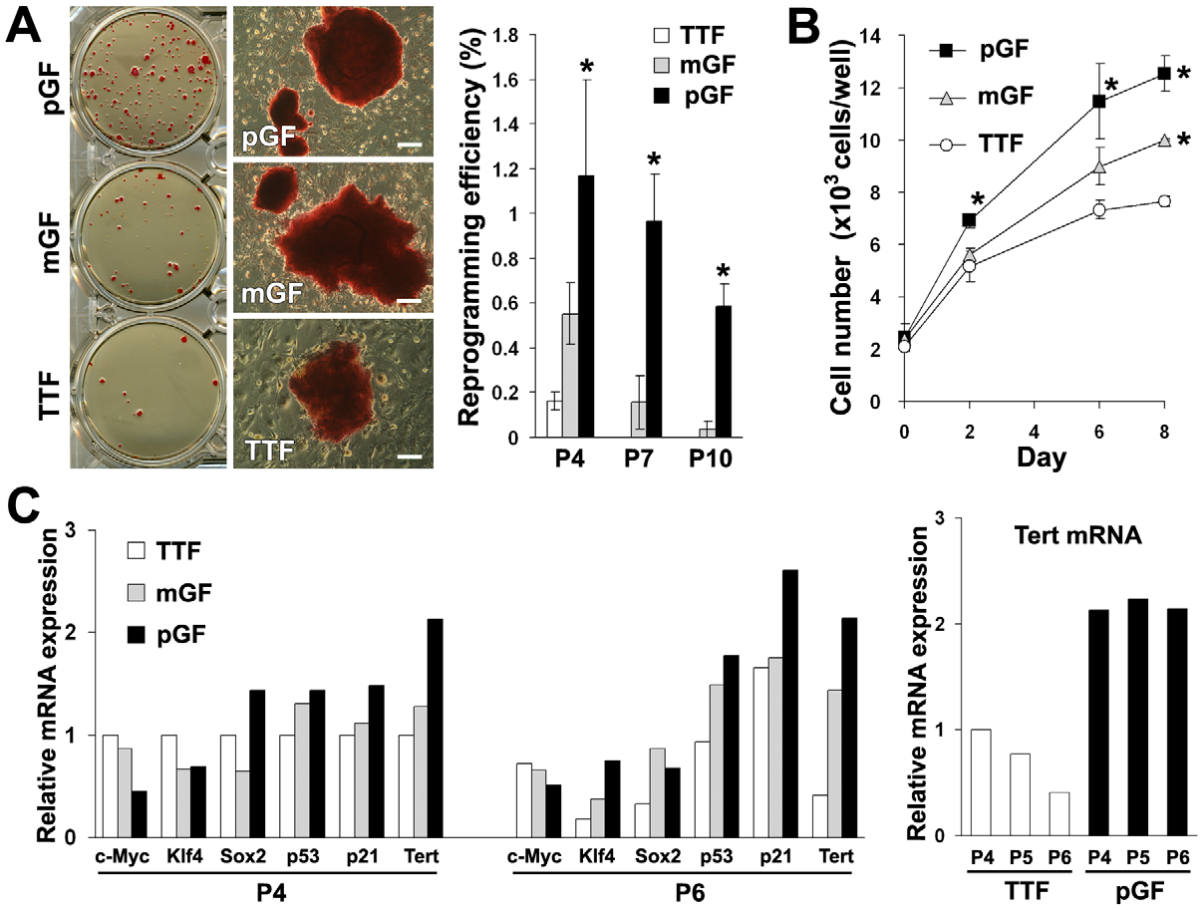
Reprogramming efficiency of mouse GF- and TTF-derived iPS cells. (A) Fibroblasts established from palatal (pGFs) and mandibular (mGFs) gingival tissues as well as tail-tips (TTFs) of the same mouse were simultaneously induced into iPS cells by four-factor transduction. Left panels: ES cell-like colony formation from each cell type at passage 4 was determined by ALP staining (Scale bars; 200 µm). Right panel: The reprogramming efficiency at passages 4, 7 and 10 was calculated as the number of ALP-stained iPS colonies formed per number of infected cells seeded. The data represent the mean values ± s.d. (n = 4). Significant differences (*P<0.01: ANOVA with Dunnett's correction for multiple comparisons) were evaluated with respect to the values for TTF at each passage number of cultures. (B) A cell proliferation assay was performed on the pGF, mGF and TTF cultures at passage 5. The data represent the mean values ± s.d. (n = 3). Significant differences (*P<0.01: ANOVA with Dunnett's correction for multiple comparisons) were evaluated with respect to the values for TTF at each time point. (C) Real-time RT-PCR analysis for endogenous expression of c-Myc, Klf4, Sox2, p53, p21 and Tert genes in pGFs, mGFs and TTFs at passages 4 and 6 (left panel). The expression level of Tert mRNA in pGFs was maintained for 6 passages, while that in TTFs decreased as the passage number increased (right panel). Expression of GAPDH was used as an internal control. The data represent the relative mRNA expression levels with respect to the expression levels of each gene in TTFs at passage 4.

To compare the reprogramming efficiency between mouse GFs and tail-tip fibroblasts (TTFs), pGF, mGF and TTF cultures were established from the same individual mouse. The reprogramming efficiency of the pGFs, mGFs and TTFs at passage 4 was 1.2%, 0.6% and 0.1%, respectively (**Fig. 5A**). During the experimental period (4–10 passages), the reprogramming efficiency was the highest in the pGFs, followed by the mGFs, and then the TTFs. No ES cell-like colonies had emerged from the TTF cultures transduced after 7 passages, whereas pGFs transduced after 10 passages were still amendable to reprogramming, at a rate of 0.6%.

Cell proliferation assays performed on cells at passage 5 showed that the number of pGFs and mGFs on day 8 was significantly higher than that of TTFs (*P*<0.01) (**Fig. 5B**). The proliferative capacity of pGFs was maintained for at least 20 passages, while that of TTFs decreased significantly after 10 passages (data not shown). Real-time RT-PCR showed that the expression level of telomerase reverse transcriptase (Tert) mRNA in pGFs and mGFs was maintained for 6 passages, while that in TTFs decreased as the passage number increased (**Fig. 5C**). Similar expression levels were detected for c-Myc, Klf4, Sox2, p53 and p21 genes among the pGFs, mGFs and TTFs, although the expression levels of klf4, p53 and p21 in GFs were slightly higher than in TTFs at passage 6 (**Fig. 5C**). Expression of Oct3/4 mRNA was not detected in the primary GFs and TTFs.

### Induction of Human Gingival Fibroblast (hGF)-Derived iPS Cells

**Figure 6:**
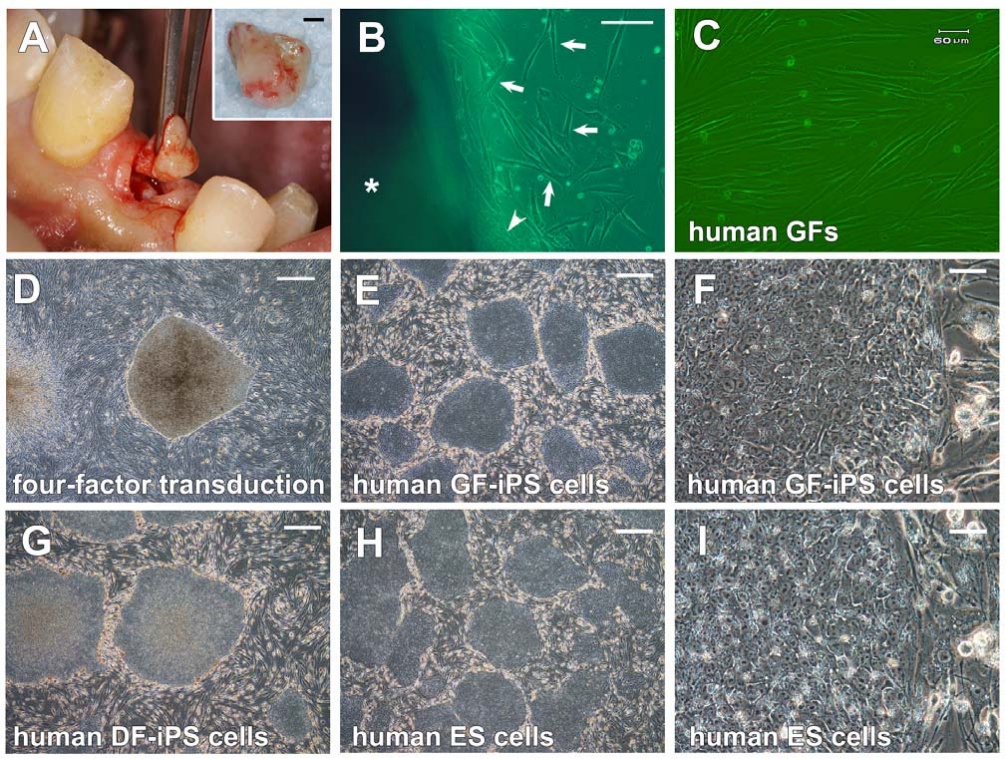
Generation of human GF-derived iPS cells. (A) Resection of gingival tissue from the adult patient during dental implant surgery. The resected gingival tissue (inset: scale bar; 1 mm) is generally treated as biomedical waste. (B) Fibroblasts (arrow) and epithelial cells (arrowhead) migrated out of the human gingival tissue (asterisk). Scale bar; 100 µm. (C) The morphology of established hGFs (Passage 4). Scale bar; 60 µm. (D) The morphology of a colony derived from hGFs 26 days after transduction of the four factors. Scale bar; 500 µm. (E–I) Morphology of (E and F) clonal hGF-derived iPS cells (clone 547A-1), (G) human dermal fibroblast-derived iPS cells (DF-iPS cells) and (H and I) human KhES-3 ES cells. Scale bars; 500 µm for E, G and H, and 50 µm for F and I.

When gingival tissues from the patient (**Fig. 6A**) were cultured on a gelatin-coated dish, fibroblasts and epithelial cells proliferated out of the tissues (**Fig. 6B**). Homogeneous fibroblast culture was established in serum- and calcium- containing media (**Fig. 6C**). Four-factor-transduced cells yielded iPS cell-like colonies (**Fig. 6D**); these colonies were picked mechanically and five clone cultures (hGF-iPS-547A-1 to -5) were established (**Fig. 6E, 6F**). The colonies could be expanded and displayed the same morphology and growth characteristics as colonies of iPS cells obtained from human dermal fibroblasts (**Fig. 6G**) and human ES cells (**Fig. 6H, 6I**).

### Characteristics and Differentiation of the hGF-iPS Cells

**Figure 7:**
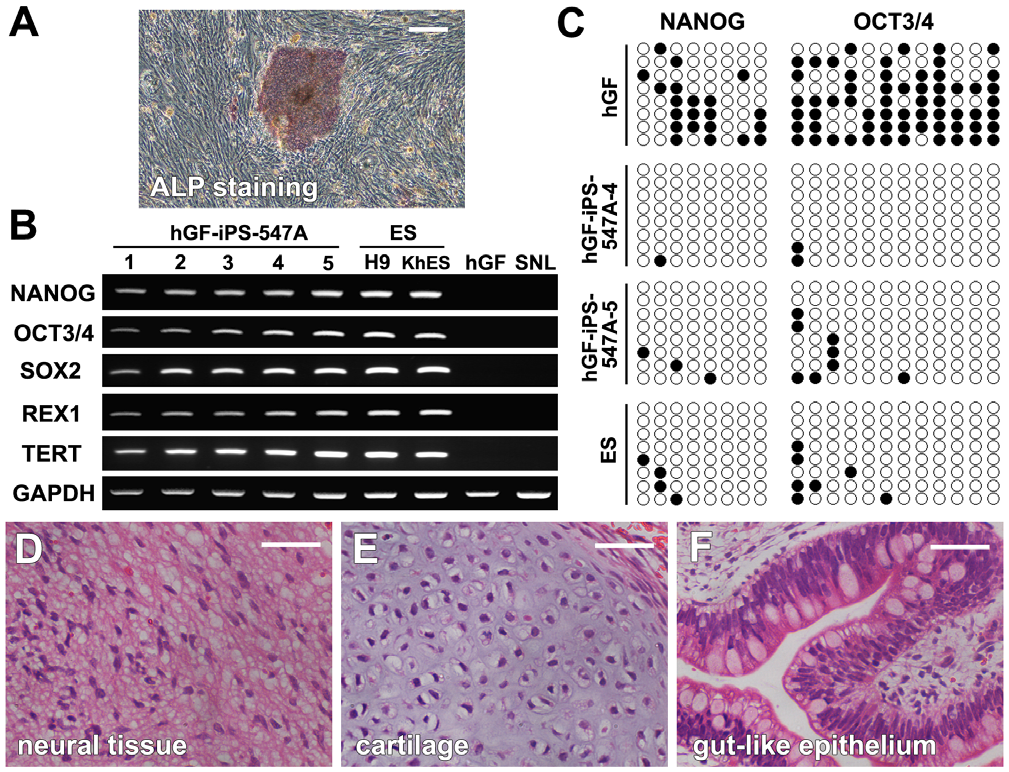
Characteristics and differentiation of the hGF-iPS cells. (A) hGF-iPS cell colonies positively stained for ALP (clone 547A-1). Scale bar; 300 µm. (B) RT-PCR analysis of ES cell specific genes (NANOG, REX1, TERT, endogenous OCT3/4 and SOX2) in hGF-iPS clones, human ES cell lines (H9 and KhES-3), parental hGFs and SNL feeder cells. GAPDH was used as a loading control. (C) Bisulfite sequencing of the NANOG and OCT3/4 promoters demonstrated that CpGs in the parental hGFs were converted to a demethylated state in the hGF-iPS cells (clones 547A-4 and -5), resulting in a methylation pattern similar to that of human H9 ES cells. (D–F) H&E staining of teratoma sections showed differentiation of hGF-iPS-547A-3 cells into neural tissues (D: ectoderm), cartilage (E: mesoderm), and gut-like epithelial tissues (F: endoderm). Scale bars; 50 µm.

The colonies of all generated hGF-iPS cell clones stained positively for ALP activity (**Fig. 7A**). RT-PCR analysis showed that all hGF-iPS cell clones expressed ES cell specific genes, including NANOG, REX1, TERT, endogenous OCT3/4 and SOX2, at levels comparable to those in H9 and KhES3 human ES cell lines (**Fig. 7B**). In contrast, these genes were not expressed in parental hGFs or SNLP feeder cells. Bisulfite genomic sequencing revealed the percentage methylation of CpGs in the NANOG promoter regions of parental hGF, hGF-iPS (average of 2 clones) and H9 human ES cells to be 32.8%, 3.2% and 6.3%, respectively. The respective percentages for OCT3/4 in hGF, hGF-iPS (average of 2 clones) and H9 human ES cells were 64.6%, 5.2% and 7.3% (**Fig. 7C**).

Cloned hGF-iPS-547A-2 and -3 cells formed teratomas after injection into SCID mouse testes. Histological examination of the teratomas at week nine after injection revealed representative tissues originating from the three embryonic germ layers, including ectodermal neural tissues, mesodermal cartilage and endodermal gut-like epithelial tissues (**Fig. 7D–F**). Therefore, like human ES cells, the hGF-iPS cells generated in this study had the capacity to differentiate into tissues representative of the three germ layers *in vivo*.

## Discussion

Tissue engineering become a new frontier in dentistry for, among other applications, regeneration of missing oral tissues [28]. However, engineering applications for tooth, jawbone, temporomandibular joint cartilage and periodontal tissues await the establishment of a stem cell source that allows easy collection by dentists [29]. In view of the potential clinical applications of iPS cells, various types of discarded or easily obtainable normal human tissues, especially those that can be obtained with minimal patient discomfort such as peripheral blood [30], should be considered as potential sources of iPS cells. From a practical standpoint, the complicated cell isolation process, low numbers of isolated cells and slow proliferation necessitate a long-term *ex vivo* expansion step for obtaining sufficient cells for iPS induction. Such a step is costly, time-consuming, and increases the risk of cell contamination and loss.

Several sources have shown more efficient iPS cell generation, such as human keratinocytes from hair follicles or epidermal biopsies [31] as well as mesenchymal stem cells of dental origin from dental pulp [32], [33] and impacted third molars [33], [34]. However, there are several practical limitations to using these cells for tissue engineering. For example, expansion of keratinocytes requires serum-free low-calcium medium, which is relatively costly, to prevent terminal differentiation. Isolation of the dental stem cells may not be sufficiently convenient to allow easy harvesting whenever the cells are needed because it requires tooth or pulp extraction surgery, and the missing tissues can not regenerate. In addition, culture of these non-terminally differentiated cells requires a high level of skill to adequately maintain cellular homogeneity. Similar limitations apply to many other possible sources of iPS cells. An ideal autologous source should thus allow easy collection of a large number of cells that can be grown in a simple culture system, and that can quickly be cultured to quantities sufficient to obviate extensive and long-term expansion. Oral gingival tissue may represent such a source. In this study, GFs from both mouse and human gingival tissues were easily established.

The mandibular mucosa of a 10-week-old mouse is too small to extract only gingival tissues. Therefore, muscle and bone tissues around the mandibular gingival tissues were carefully removed, and mGFs were obtained from the outgrowth of fibroblastic cells from the remaining tissues. This technical limitation may have resulted in the possible contamination of mGFs with some myoblasts or bone marrow stromal fibroblasts. On the other hand, the mouse palatal mucosa was easily extracted en bloc without any contamination by surrounding tissues (**Fig. 1A**). Therefore, the pGFs used in this study may have been more homogeneous than the mGFs. Nevertheless, both types of adult mouse primary GFs proliferated well and could be successfully reprogrammed into iPS cells that differentiated into cells and tissues representing all three germ layers *in vitro* and *in vivo*.

All reprogramming approaches investigated to date seem to involve epigenomic modification. GF-iPS-3F showed a greater decrease in the CpG methylation ratio in the promoter regions of Nanog and Oct3/4 in comparison to GF-iPS-4F cells that resulted in a methylation pattern similar to that of mouse ES cells. Nakagawa *et al.* [35] reported that the omission of c-Myc transduction resulted in the generation of high-quality iPS cells from mouse TTFs, in which Nanog is strongly activated and the retroviruses are silenced. Consistently, our results showed that GF-iPS-3F cell clones highly expressed ES cell marker genes, including Nanog and endogenous Oct3/4. On the other hand, GF-iPS-4F cell clones expressed these genes at lower levels than in mouse ES cells and showed partial DNA methylation in restricted areas of the promoters. These results may in part be due to incomplete reprogramming effects of four-factor transduction during GF-iPS-4F cell induction in our system, which did not utilize a specific system for reprogrammed cell selection (e.g., drug selection and mice genetically modified for Nanog expression [36]). Because incompletely reprogrammed GF-iPS-4F cells at least possess multipotency, they might be used in some tissue regenerative approaches; however, concerns remain that reactivation of the c-Myc oncogene in iPS cells could increase tumorigenicity, thereby hindering potential clinical applications [35].

c-Myc has recently been shown to be dispensable for direct reprogramming [35], [37]. It is conceivable that one major function of c-Myc is to enhance proliferation, thereby accelerating the reprogramming process, possibly by increasing the speed of stochastic events that lead to the formation of iPS cells [37]. Consistently, the three factors without c-Myc were able to initiate a slower reprogramming process that was sufficient to fully reprogram mouse GFs after a longer time period. Indeed, GF-iPS-3F cells demonstrated almost complete DNA demethylation in the promoter regions of Nanog and Oct3/4. In addition to the specific induction, the long reprogramming time course in GF-iPS-3F cells may be responsible for the low level of methylation in the promoters. All five tested GF-iPS-3F clones readily produced viable chimeric newborn and adult mice. Moreover, mGF-iPS-3F-3 cells contributed to germline transmission, which definitively demonstrates that they were pluripotent and functionally indistinguishable from ES cells. So far, 4 out of the 78 mGF-iPS-3F cell chimeras (5%) produced in the study died within six months; however, apparent tumors were not observed in the dead mice. These results suggest that high-quality iPS cells can be generated from adult mouse GFs by transduction of the three factors (Oct3/4, Sox2 and Klf4) without any specific system for the selection of reprogrammed cells. However, because Klf4, the remaining oncogenic factor, or insertional mutagenesis due to retroviral transduction itself might also cause tumor formation, it will be important to investigate the possibility of using recombinant proteins (and small molecules) to reduce the number of genetically transduced factors required for iPS cell induction, or even to entirely obviate the need for viral gene delivery.

TTFs were the first type of adult cells to be reprogrammed into iPS cells [1]. Since then, other adult cell types with the potential for easier reprogramming have been tested [10], [14]. Our results show that pGFs can be reprogrammed to pluripotency at least 7-fold more efficiently than TTFs at the same passage number derived from the same mouse. In addition, GFs, especially pGFs, maintained their high reprogramming efficiency for at least ten passages. On the other hand, no-ES cell-like colonies emerged from TTFs transduced after 7 passages, possibly due to replicative senescence [38]. The lower reprogramming efficiency of mGFs compared to pGFs in our system may have been due to the establishment of a heterogeneous cell population in the mGFs.

It was initially speculated that the high efficiency of iPS cell generation from GFs might have been due to high endogenous expression levels of at least one of the four defined pluripotency-inducing factors, or due to reduced activation of the p53 pathway [39], [40]. However, no significant differences in the endogenous expression of the four factor genes or of p53 or p21 were detected. On the other hand, GFs showed significantly greater proliferation in comparison to TTFs. Additionally, pGFs proliferated well for at least 20 passages, while the proliferation of TTFs decreased after 10 passages (data not shown). Moreover, GFs consistently showed higher expression of Tert mRNA compared to TTFs. Tert is one of the major subunits in the telomerase complex [41], and the transcription of Tert gene correlates with telomerase activity in most cells [42]. The high expression of the Tert gene in GFs may therefore explain their high proliferative capacity. The high proliferative capacity of the GFs should be advantageous for retroviral integration due to increased likelihood of cell division during transduction. The higher efficiency of GF reprogramming, therefore, may at least partially be due to the high proliferation rate of the GFs. Wounds in the oral mucosa show faster closure with less scar formation than skin wounds [16], [17], partly because oral GFs differentially express early wound closure-related genes, such as FGFR1OP2/wit3.0 [18], [19]. It is therefore possible that intrinsic differences in gene expression patterns between the GFs and TTFs may also underlie differences in reprogramming efficiency.

We also demonstrated that iPS cells could be generated from human gingival tissues, which underscores the potential value of this promising cell source for human applications. When we transduced human dermal fibroblasts (HDF1388 [5]) in parallel with human GFs under the same experimental setting and infection protocols, fewer ES cell-like colonies emerged from the dermal fibroblasts, suggesting that human GFs might be more readily reprogrammed into iPS cells. Yan *et al.* [33] recently reported that dental stem cells can be efficiently reprogrammed into iPS cells by lentiviral transduction of LIN28/NANOG/OCT4/SOX2 and by retroviral transduction of c-MYC/KLF4/OCT4/SOX2. However, their protocol did not generate ES cell-like colonies from human gingival fibroblasts and foreskin fibroblasts. They also indicated that dental stem cells express a number of ES cell-associated genes, thus suggesting that these stem cells have epigenetic advantages for reprogramming. Therefore, the reprogramming efficiency of terminally differentiated GFs may be inferior to that of undifferentiated stem cells.

It is unknown at present whether iPS cells derived from different types of cells behave in the same manner [33]. Specifically, iPS cells from different cell types may differ in their ability to undergo guided differentiation [43]. Therefore, GF-iPS cells should be further characterized and compared to ES cells and iPS cells derived from other sources. Potential differences in the reprogramming efficiency between cells isolated from humans and mice also remain to be elucidated. Nonetheless, the high replication capacity of GFs should permit not only the generation of sufficient cells for iPS cell induction, but also the efficient generation of iPS cells from multiple expanded cell cultures. The intrinsic features of GFs from easily obtainable gingival tissues could be of benefit for regenerative medicine and drug screening, especially in dentistry, as it is easy for dental associates to establish primary cell cultures with minimal patient discomfort. Additionally, establishment of iPS cell banks with various human leukocyte antigen (HLA) types should be useful for general regenerative medicine, as the establishment of clinical-grade iPS cell lines from individual patients would require much time and high cost [32]. Collection of gingiva considered until now to be biomedical waste from healthy volunteers and efficient iPS cell generation from this tissue may allow the development of a cell banking system for a wide range of medical applications.

In conclusion, the efficient reprogramming of mouse gingival fibroblasts to pluripotency is expected to provide a valuable experimental model for investigating the basis of cell source-dependent cellular reprogramming and pluripotency, which may thus lead to a practical alternative for the generation of patient- and disease-specific pluripotent stem cells.

## Materials and Methods

### Ethics Statement

All animal experiments in this study strictly followed a protocol approved by the Institutional Animal Care and Use Committee of Osaka University Graduate School of Dentistry (approval number: 20-009). Written approval for human gingival tissue collection and subsequent iPS cell generation and genome/gene analyses performed in this study was obtained from the Institutional Review Board at Osaka University Graduate School of Dentistry (approval number: H21-E7) and the Ethics Committee for Human Genome/Gene Analysis Research at Osaka University (approval number: 233), and written informed consent was obtained from each individual participant.

### Cell Culture

Mouse GF cultures were established from either pGFs or mGFs obtained from 10-week-old adult male C57BL/6J mice. To establish TTF cultures, tails from mice were peeled and minced into 1-cm pieces [38]. The extracted palatal and molar mucosal tissues or minced tails were placed on a 0.1% gelatin-coated 30-mm tissue culture dish and maintained in MF-start medium (Toyobo, Osaka, Japan) at 37°C with 5% CO_2_. When fibroblasts migrated out of the tissues (**Fig. 1B**), the tissues were removed. When the cells reached subconfluence, they were harvested and transferred to a gelatin-coated 60-mm tissue culture dish (Passage 1) and cultured in “fibroblasts and Platinum-E (FP) medium”, which consists of Dulbecco's Modified Eagle's Medium (DMEM without sodium pyruvate; Nacalai Tesque, Kyoto, Japan), 10% fetal bovine serum (FBS; Sigma, St. Louis, MO), 50 units/ml penicillin, and 50 µg/ml streptomycin (Nacalai Tesque). Culture in serum- and calcium-containing media favorably selects fibroblasts from heterogeneous populations of migrating epithelial cells and fibroblasts in the mucosal tissues [44].

Primary hGF cultures were established according to the previously described protocol [44] from healthy gingival tissues discarded during surgery on a 24-year-old man.

SNLP76.7-4 feeder cells and the mouse ES cell line (AB2.2) were graciously supplied by Dr. Allan Bradley of the Sanger Institute (London, UK). Platinum-E packaging cells [45] for retrovirus production were graciously supplied by Dr. Toshio Kitamura (University of Tokyo, Japan). Human ES cell line H9 was obtained from WiCell™ Research Institute (Wilmington, MA), and human ES cell line KhES-3 and human dermal fibroblast-derived iPS cells [5] were obtained from Institute for Frontier Medical Sciences, Kyoto University. The human ES cells were treated according to the guidelines for utilization of human ES cells established by the Ministry of Education, Culture, Sports, Science and Technology, Japan.

### Retrovirus Production

Moloney murine leukemia virus (MMLV)-based retroviral vectors (pMXs-IRES-puro) containing murine and human c-Myc, Oct3/4, Sox2 or Klf4 cDNA were obtained from Addgene (Cambridge, MA), and the pMX-GFP retroviral vector was purchased from Cell Biolabs (San Diego, CA). Nine micrograms of each plasmid vector were separately added to tubes containing Opti-MEM-I mediun (Invitrogen) and FuGENE 6 transfection reagent (Roche, Basel, Switzerland); each plasmid was then separately transfected into 100-mm dishes containing Platinum-E packaging cells [38]. The transfection efficiency was monitored by evaluation of GFP expression under a fluorescence microscope. The efficiency of transfection into Plat-E cells was typically >60%, as indicated by GFP expression. The next day, the culture medium was exchanged for fresh FP medium. After 24 hours, the virus-containing supernatants were mixed together and used for retroviral transduction.

### Induction of iPS Cells

Twenty-four hours before transduction, mouse pGFs and mGFs (5 passages) were seeded at 5×10^5^ cells per 100-mm dish in FP medium containing 3 ng/ml bFGF (Peprotech, London, UK). For the four-factor transduction, supernatants with retroviruses coding c-Myc, Oct3/4, Sox2, Klf4 and GFP were mixed at a ratio of 1∶1∶1∶1∶3. When the mGFs were transduced with only three factors, the supernatants containing retroviruses coding Oct3/4, Sox2, Klf4 and GFP were mixed at a ratio of 1∶1∶1∶3. The cells were incubated overnight in the virus/polybrene (4 µg/ml)/bFGF (10 ng/ml)-containing supernatants. On days one and three after transduction, the culture medium was exchanged for fresh FP medium containing 3 ng/ml bFGF.

At four days after transduction, the cells in the culture dishes were re-seeded onto 60-mm dishes at 0.1–1×10^3^ cells/cm^2^ for the four-factor transduction, and onto 100-mm dishes at 0.7–1×10^4^ cells/cm^2^ for three-factor transduction; mitomycin C-inactivated SNLP76.7-4 cells were used as a feeder layer. The next day, the culture medium was exchanged for “ES medium”, which consisted of DMEM, 15% FBS, 2 mM L-glutamine, 1×10^−4^ M nonessential amino acids, 1×10^−4^ M 2-mercaptoethanol, 50 U penicillin, and 50 µg/ml streptomycin. The medium was changed every day. The three-factor-infected GFs were harvested with deteriorated feeder cells at 30 days after transduction and then were re-seeded onto a new feeder layer.

The colonies demonstrating minimal GFP expression were identified and mechanically picked for expansion. After expansion, clonal colonies showing ES cell-like proliferation and morphology, including a round shape, large nucleoli and scant cytoplasm, were selected for establishing clonal iPS cell cultures.

To demonstrate the expression of the ES cell marker ALP in GF-derived iPS cell colonies, a standard ALP staining protocol was used [46]. TEM was used to determine the structure of individual cells from the GF-derived iPS cell colonies. The colonies were fixed first with 1% paraformaldehyde and 1.25% glutaraldehyde in phosphate-buffered saline (PBS), and second with 2% osmium tetroxide. The fixed colonies were then embedded in epoxy resin and stained with methylene blue, followed by 1-µm sectioning of the resin for optical microscopy. Additionally, 70-nm sections were stained with 2% uranyl acetate and Sato's lead stain, and examined using a JEM 1200EX (JOEL, Tokyo, Japan) operated at 80 kV for TEM.

Induction of iPS cells from primary hGFs (7 passages) via introduction of four factors was performed using a previously described protocol [47]. According to this protocol, hGFs were first infected with a lentivirus to express the mouse Slc7a1 gene (by a plasmid vector from Addgene) and then infected with retroviruses coding human c-MYC, OCT3/4, SOX2 and KLF4 genes for iPS cell induction.

### RT-PCR Analysis

Total RNA derived from mouse or human clonal GF-derived iPS or ES cell colonies was used for RT-PCR analysis. Total RNA was extracted with an RNeasy Mini Kit (QIAGEN, Hilden, Germany). After DNase I treatment (Ambion, Austin, TX), cDNA was synthesized from 1 µg of total RNA using Super Script III reverse transcriptase (Invitogen, Carlsbad, CA). The cDNA target was amplified by PCR using Taq DNA polymerase (Promega, Madison, WI) following the manufacturer's recommendations. The primer pairs used are given in **Table S1**. PCR products were subjected to 1.5% agarose gel electrophoresis with ethidium bromide staining and visualized under ultraviolet light illumination. The expression of glyceraldehyde-3-phosphate dehydrogenase (GAPDH) mRNA was used as an internal control.

### *In vitro* Differentiation of Mouse GF-Derived iPS Cells

To determine the differentiation ability of GF-derived iPS cells *in vitro*, we used floating cultivation to form EBs [48]. For EB formation, mouse GF-derived iPS cells were harvested by trypsinization and transferred to low-attachment bacterial culture dishes in the ES medium. After 3 days of floating cultivation to form EBs, aggregated cells were plated onto gelatin-coated 8-well glass chamber slides (Nalge Nunc International, Naperville, IL) or 12-well tissue culture plates, and incubated in ES medium. The culture medium was changed twice a week.

For immunocytochemistry, cells cultured in glass chamber slides for 3 to 10 days after expansion were fixed in 10% buffered formalin phosphate (Wako, Osaka, Japan) and incubated in 1% bovine serum albumin and 0.1% Triton-X100 in PBS for 20 min. After two washes in PBS, the cells were incubated with a mouse anti-human α-SMA monoclonal antibody (0.05 mol/L; clone 1A4, Dako, Glostrup, Denmark) or rabbit anti-human AFP polyclonal antibody (0.05 mol/L; Dako) for 30 min at room temperature, or a mouse anti-human β-III tubulin monoclonal antibody (0.5 µg/ml; clone TU-20, Millipore, Temecula, CA) or control IgG (0.5 µg/ml; mouse IgG whole molecules: Santa Cruz Biotechnology, Santa Cruz, CA) overnight at 4°C [1]. The cells were then washed and incubated for 30 min at 37°C with Alexa 488 (green dye) or 568 (red dye) conjugated to goat anti-mouse or anti-rabbit IgG (1∶500; Molecular Probes, Eugene, OR), followed by 4,6′-diamidino-2-phenylindole (DAPI: Roche) nuclear staining.

For osteogenic cell detection, EBs cultured in gelatin-coated 12-well tissue culture plates in ES medium for 30 days were stained using a standard von Kossa staining method [49] to demonstrate the extent of nodule mineralization.

### Bisulfite Genomic Sequencing

Genomic DNA was isolated from the aggregated mouse GF-derived iPS cells and ES cells floating for 3 days or from the attached human GF-derived iPS cells and H9 ES cells harvested by CTK solution [47]. Information about the promoter regions and CpG loci of Nanog and Oct3/4 was obtained from a previous study [5] and the Data Base of Transcriptional Start Sites (DBTSS Ver. 7.0: http://dbtss.hgc.jp/). Bisulfite treatment was performed using the EpiTect Bisulfite kit (Qiagen) according to the manufacturer's recommendations. Bisulfite PCR primers [1], [5] are listed in **Table S1**. Amplified products were cloned into the pGEM-T Easy Vector (Promega). Five to eight randomly selected clones were sequenced with the SP6 forward and reverse primers for each gene.

### Teratoma Formation and Histological Analysis

Eight-week-old immunodeficient mice (C.B-17 SCID; Clea Japan, Tokyo, Japan) were anesthetized with diethyl ether and an intraperitoneal injection (0.1 ml per 100 g body weight) of a 10× dilution of Nembutal (Dainippon Sumitomo Pharmaceutical, Osaka, Japan). Twenty microliters of a mouse or human GF-derived cell suspension (0.2–0.5×10^6^ cells/testis) in cold Hank's balanced salt solution or DMEM/F12 (Invitrogen) were injected into the medulla of mouse testes using a Hamilton syringe. The mice were thereafter housed with free access to water and food under specific pathogen-free conditions. After 7–10 weeks, the teratomas were excised after perfusion with PBS followed by a fixative solution containing 1% paraformaldehyde and 1.25% glutaraldehyde, and subjected to histological analysis. Specimens were embedded in paraffin, and sectioned at 3 µm for hematoxylin and eosin (H&E) staining.

### Chimera Formation

Superovulation [intraperitoneal administration of 5 I.U. pregnant mare serum gonadotropin (PMSG) followed after 48 hr by 5 I.U. human chorionic gonadotropin (hCG)] was induced in eight-week-old female mice [Jcl:MCH (ICR), CREA Japan], which were then mated with Jcl:MCH (ICR) males. Embryos at the 2-cell stage were collected at day 1.5 after vaginal plug observation and flushed in M2 medium (Sigma). Embryos were then cultured in KSOM culture medium (Chemicon) in the incubator (37°C, 5% CO_2_ in air) until they became blastocysts.

Mouse GF-derived iPS cells were harvested using 0.25% trypsin to obtain a single cell suspension. Single cells were then transferred into the micromanipulation chamber in a drop of DMEM medium containing 10% fetal calf serum and 15 mM HEPES. Groups of 20 to 25 cells were injected into each single blastocyst. The injected embryos were then transplanted into 2.5 dpc pseudopregnant Jcl:ICR recipient females. Chimeric male mice were mated with female mice [Jcl:MCH (ICR)] to validate germline transmission.

### Determination of Reprogramming Efficiency

TTF, pGF and mGF cultures were established from the same individual mouse (10 weeks of age). Four-factor transduction (without GFP) was performed using cell cultures with identical passage numbers. The transduction of each cell type was performed simultaneously using the same virus-containing supernatants. The cell cultures used for the comparisons were between passage numbers four and ten. Each cell culture was then seeded in 6-well tissue culture plates (1×10^4^ cells/well) with the feeder cells. iPS cell colonies were identified based on ES cell-like morphology, and ALP staining was used to facilitate their identification. The reprogramming efficiency was calculated as the number of iPS colonies formed per number of transduced cells seeded.

A cell proliferation assay was performed on pGF, mGF and TTF cultures with identical passage numbers. The cells were seeded in 96-well tissue culture plates (2×10^3^ cells per well) and maintained in FP medium. The culture medium was renewed every other day. The number of cells was evaluated using the WST-1 cell counting assay (Dojindo Laboratories, Kumamoto, Japan) as described previously [50].

The endogenous mRNA expression of Oct3/4, Sox2, Klf4, c-Myc, p53, p21 and Tert in pGFs, mGFs and TTFs at passages 4 to 6 was determined by real-time RT-PCR analysis. TaqMan primer and probe sets used are Mm00488369_sl (Sox2), Mm00516105_gl (Klf4), Mm00487804_ml (c-Myc), Mm00441964_g1 (p53), Mm00432448_m1 (p21), Mm00436931_m1 (Tert) and 4352339E (GAPDH).

## Supporting Information

Table S1 Primers used for RT-PCR and bisulfite genomic sequencing analyses.
(0.07 MB DOC)

Movie S1 Beating cardiomyocytes observed following spontaneous differentiation of pGF-iPS-4F-3 cells.
(1.53 MB WMV)
