# Supplementary material for: Gingival Fibroblasts as a Promising Source of Induced Pluripotent Stem Cells
Source: PLoS One. 2010 Sep 14;5(9):e12743. doi: 10.1371/journal.pone.0012743 (PMC2939066; doi:10.1371/journal.pone.0012743)
Supplement: Table S1 — Primers used for RT-PCR and bisulfite genomic sequencing analyses. (0.07 MB DOC) [file pone.0012743.s001.doc]

**Supporting Information**

**Table S1**: Primers used for RT-PCR and bisulfite genomic sequencing analyses

| **gene** | **species** | **primers** (Fw, forward; Rv, reverse) | **product size** |
| --- | --- | --- | --- |
| Nanog | mouse | Fw: 5’-AGG GTC TGC TAC TGA GAT GCT CTG-3’  Rv: 5’-CAA CCA CTG GTT TTT CTG CCA CCG-3’ | 364 bp |
| ERas | mouse | Fw: 5’-ACT GCC CCT CAT CAG ACT GCT ACT-3’  Rv: 5’-CAC TGC CTT GTA CTC GGG TAG CTG-3’ | 210 bp |
| Rex1 (Zfp42) | mouse | Fw: 5’-ACG AGT GGC AGT TTC TTC TTG GGA-3’  Rv: 5’-TAT GAC TCA CTT CCA GGG GGC ACT-3’ | 290 bp |
| Oct3/4  (endogenous) | mouse | Fw: 5’-TCT TTC CAC CAG GCC CCC GGC TC-3’  Rv: 5’-TGC GGG CGG ACA TGG GGA GAT CC-3’ | 224 bp |
| Sox2  (endogenous) | mouse | Fw: 5’-TAG AGC TAG ACT CCG GGC GAT GA-3’  Rv: 5’-TTG CCT TAA ACA AGA CCA CGA AA-3’ | 297 bp |
| Klf4  (endogenous) | mouse | Fw: 5’-GCG AAC TCA CAC AGG CGA GAA ACC-3’  Rv: 5’-TCG CTT CCT CTT CCT CCG ACA CA-3’ | 709 bp |
| c-Myc  (endogenous) | mouse | Fw: 5’-TGA CCT AAC TCG AGG AGG AGC TGG AAT C-3’  Rv: 5’-AAG TTT GAG GCA GTT AAA ATT ATG GCT GAA GC-3’ | 170 bp |
| GAPDH | mouse | Fw: 5’-CAC CAT GGA GAA GGC CGG GG-3’  Rv: 5’-GAC GGA CAC ATT GGG GGT AG-3’ | 418 bp |
| Methylation analysis of Oct3/4 | mouse | Fw: 5’-GGT TTT TTA GAG GAT GGT TGA GTG-3’  Rv: 5’-TCC AAC CCT ACT AAC CCA TCA CC-3’ | 367 bp |
| Methylation analysis of Nanog | mouse | Fw: 5’-GAT TTT GTA GGT GGG ATT AAT TGT GAA TTT-3’  Rv: 5’-ACC AAA AAA ACC CAC ACT CAT ATC AAT ATA-3’ | 474 bp |
| NANOG | human | Fw: 5’-CAG CCC CGA TTC TTC CAC CAG TCC C-3’  Rv: 5’-CGG AAG ATT CCC AGT CGG GTT CAC C-3’ | 391 bp |
| REX1 | human | Fw: 5’-CAG ATC CTA AAC AGC TCG CAG AAT-3’  Rv: 5’-GCG TAC GCA AAT TAA AGT CCA GA-3’ | 306 bp |
| TERT | human | Fw: 5’-CCT GCT CAA GCT GAC TCG ACA CCG TG-3’  Rv: 5’-GGA AAA GCT GGC CCT GGG GTG GAG C-3’ | 446 bp |
| OCT3/4 (endogenous) | human | Fw: 5’-GAC AGG GGG AGG GGA GGA GCT AGG-3’  Rv: 5’-CTT CCC TCC AAC CAG TTG CCC CAA AC-3’ | 144 bp |
| SOX2  (endogenous) | human | Fw: 5’-GGG AAA TGG GAG GGG TGC AAA AGA GG-3’  Rv: 5’-TTG CGT GAG TGT GGA TGG GAT TGG TG-3’ | 151 bp |
| GAPDH | human | Fw: 5’- GTC AAG GCC GAG AAT GGG AA -3’  Rv: 5’- GCT TCA CCA CCT TCT TGA TG -3’ | 613 bp |
| Methylation analysis of OCT3/4 | human | Fw: 5’-GAG GTT GGA GTA GAA GGA TTG TTT TGG TTT-3’  Rv: 5’-CCC CCC TAA CCC ATC ACC TCC ACC ACC TAA-3’ | 467 bp |
| Methylation analysis of NANOG | human | Fw: 5’-TGG TTA GGT TGG TTT TAA ATT TTT G-3’  Rv: 5’-AAC CCA CCC TTA TAA ATT CTC AAT TA-3’ | 336 bp |
